# Perceptions, acceptability, and utilization of HIV self-testing kits among young people in urban slums in Kampala

**DOI:** 10.3389/fpubh.2025.1704705

**Published:** 2026-01-14

**Authors:** Micheal C. Segawa, Sabrina B. Kitaka, Amelia M. Namiiro, Bruno Natuhwera, Praise D. Ahereza, Ivaan Pitua, Marvin Kirya, Denis Kiberu, Tom Okade, Sarah M. Najjuka, Ronald Olum

**Affiliations:** 1 College of Health Sciences, Makerere University, Kampala, Uganda; 2 School of Public Health, Makerere University, Kampala, Uganda

**Keywords:** HIV prevention, HIV self-testing, public health equity, self-care interventions, unplanned settlement health, youth health

## Abstract

Globally, 14% of people with HIV remain undiagnosed, delaying care and prevention. Youth in urban unplanned settlements face heightened risk due to poor access, stigma, and structural barriers, with HIV prevalence twice that of other urban areas. HIV self-testing (HIVST) offers a private, convenient approach to close this gap. We conducted a mixed-methods cross-sectional study among young people aged 15–24 living in Katanga Slum, Kampala, recruited by simple random sampling between February and May 2024. Quantitative data from 267 participants (mean age 20.1 years; 62.9% male) were analyzed using descriptive statistics and logistic regression, and qualitative data from focus group discussions were thematically analyzed. Acceptability of HIVST was high (87.6%) while utilization was lower (43.1%). Participants viewed self-testing positively, citing convenience, privacy, accuracy, stigma reduction, and clarity of instructions. Acceptability was associated with marital status, peer discussion, family support, and absence of financial barriers, while utilization was linked to religion and peer discussion. Qualitative findings highlighted privacy, affordability, availability, and trust in results as critical drivers of acceptability and use. Despite strong acceptance, uptake remains limited by cost and accessibility. Peer-led outreach, community engagement, and subsidized kits could enhance use and contribute to improved HIV prevention and early diagnosis in this high-risk population.

## Introduction

In African cities, HIV prevalence in urban unplanned settlements (“slums”) is estimated to be twice as high as in other urban areas of the same cities, yet stigma continues to undermine efforts to seek HIV prevention services, undergo testing, and initiate or adhere to treatment (1, 2). HIV self-testing (HIVST) has emerged as a promising strategy to address these gaps in testing coverage and serve as an entry point to HIV prevention services (3). Despite being a gateway to HIV care, only 89% of people living with HIV in Uganda were aware of their status by 2021, falling short of the UNAIDS 95% 20,230 target (1, 4).

HIVST has been associated with increased HIV testing uptake, particularly among high-risk and underserved populations such as young people and first-time testers. It empowers users, which helps to normalize testing (3, 5–7). According to Choko et al. (8), participants who chose not to use HIVST were noticeably more likely to worry about having HIV. Perceived facilitators of the uptake of HIVST were autonomy and self-empowerment, privacy, confidentiality, convenience, opportunity to test, including couples’ HIV testing, and ease of use. The perceived barriers included the cost of self-test kits, perceived unreliability of test results, low literacy, fear and anxiety of a positive test result, and potential psychological and social harm, as echoed by a study among young adults in Namibia, who further stressed issues with the regulation of HIVST and abuse by family members and employers (9, 10).

In Peru, men who have sex with men (MSM) found HIVST with a blood-based assay highly acceptable and feasible, while female sex workers in Botswana preferred facility-based services and a blood test over HIVST due to inadequate post-test counseling and a lack of assistance associated with HIVST (11, 12). Some HIVST kit users preferred the blood-based kit for higher accuracy, while the oral-fluid-based kit was liked for its ease of use, being painless, and being less invasive as compared to the finger-prick/whole blood-based HIVST kits (9). HIVST kit instructions were considered to be easy to read and perform, with some variations by test type, and the majority of users felt confident in their ability to read their results (13).

Despite growing literature on HIVST, limited evidence exists on the perceptions, acceptability, and utilization of self-testing kits among young people living in urban unplanned settlements in Kampala. Our research focuses on “Katanga Slum,” a settlement in the city, and similar areas. We recognize that the term “slum” carries pejorative connotations and can be stigmatizing. However, for the purpose of accurate geographical reference to this established place name and comparable communities, we use it here while emphasizing the importance of respecting the community living within it. This study aimed to explore these dimensions to inform context-specific, evidence-based strategies that can increase HIV testing uptake, promote early diagnosis, and reduce HIV transmission in resource-limited urban settings.

## Methods

### Study design

We used a cross-sectional mixed-methods study. Data was collected between February and May 2024.

### Study setting

The study took place in Katanga, Kampala’s largest urban unplanned settlement, situated between Mulago National Referral Hospital and Makerere University. Home to over 20,000 residents, more than half under 14 years old (14). Katanga faces elevated HIV, TB, and HIV/TB co-infection risks. Poverty, overcrowding, poor living conditions, substance use, transactional sex, and limited access to regulated healthcare drive these. Informal employment, gender inequities, and reliance on unregulated drug shops further exacerbate vulnerabilities to HIV infection (15, 16).

### Study population

The study population comprised young people aged 15–24 years living in urban unplanned settlements in Kampala who were present at the study site during the study period and provided informed consent.

### Sampling

#### Quantitative

Simple random sampling was used to select participants. A sampling frame of all eligible young people aged 15–24 residing in Katanga Slum was obtained with the help of local leaders, and each individual was assigned a unique number. Participants were then randomly selected using a computer-generated random number list.

#### Qualitative

Participants were selected through purposive sampling, ensuring representation across age, sex, and HIV testing experience to enrich the discussion.

### Sample size estimation

#### Quantitative

The sample size was 377, calculated using Keish and Leslie’s formula, based on a confidence level of 95% and a 5% margin of error. The estimated prevalence was adapted from a report of an average general acceptability of HIVST of 56.9% (17).

#### Qualitative

Focus group discussion (FGD) was conducted with 12 purposively selected participants, in line with accepted standards for FGD sample sizes.

### Selection criteria

#### Inclusion criteria

Young people aged 15–24 years, residing in Katanga for at least six months, and able to provide informed consent (or assent for minors with parent or guardian consent).

#### Exclusion criteria

Individuals with medical or psychological conditions that impaired their ability to participate meaningfully in the study.

### Study procedures

Quantitative data were collected using a semi-structured interviewer-administered questionnaire, covering sociodemographic details, HIV testing knowledge, perceptions of HIVST, acceptability, and utilization.

Qualitative data were obtained through a focus group discussion led by a trained facilitator using a discussion guide. The session was conducted in both English and Luganda, audio-recorded with participants’ consent, and later transcribed for analysis.

### Study variables

#### Quantitative

The dependent variables included: perception of HIV self-testing, acceptability (willingness to use an HIVST kit), and utilization (actual use of an HIV self-test kit). The independent variables comprised sociodemographic factors (age, sex, religion, marital status, income, and education), knowledge of HIVST, access to healthcare, social support (peer, family, and community influences), and prior HIV testing experience (including motivations and barriers).

#### Qualitative

Key areas of inquiry, identified from participant narratives, included perceptions of HIVST, barriers and facilitators to its use, and contextual factors shaping its acceptability and uptake.

### Data analysis

#### Quantitative

Quantitative data were entered in Excel 2019 and analyzed in STATA v16. Descriptive statistics (frequencies, proportions, means, and standard deviations) were generated. Variables associated at *p* < 0.20 in bivariate analysis and those of clinical relevance were included in the multivariable logistic regression model. Assumptions for multicollinearity, influential observations, and outliers were assessed before fitting the model; interaction and confounding were also examined. Adjusted odds ratios with 95% confidence intervals were reported, and statistical significance was set at *p* < 0.05.

#### Qualitative

Audio recordings from the focus group discussion were transcribed verbatim, then imported into ATLAS.ti (version 9) to facilitate thematic content analysis. Researchers coded meaningful data segments into initial codes, grouped related codes into broader themes, and refined them iteratively using ATLAS.ti Emerging themes were defined and illustrated using direct participant quotes.

## Results

### Quantitative

#### Socio-demographics

274 young people consented and completed the survey, yielding a response rate of 72.7%. After excluding seven records due to incomplete data, a total of 267 responses were included in the final analysis. The mean age was 20.05 years (SD = 2.60), and most participants were male (62.9%). Over half had attained secondary education (55.8%), and 58.8% had lived in the Katanga Slum for more than 5 years. The majority had never been married (81.7%) and reported a low monthly income (60.3%). The most common religious affiliation was Christianity (70.0%) (Table 1).

**Table 1:** Socio-demographic characteristics of young people in the Katanga Slum.

Variable	Frequency (*n* = 267)	Percentage (%)
Age	Mean (SD) = 20.05 ± 2.60	
Gender		
Female	99	37.1
Male	168	62.9
Level of education		
Never been educated	3	1.1
Primary	108	40.5
Secondary	149	55.8
Tertiary	7	2.6
Duration of stay in the slum		
≤ 5 years	110	41.2
≥ 5 years	157	58.8
Marital status		
Never married	218	81.7
Ever been married	49	18.3
Level of monthly income		
Low (< UGX100,000 ≈ 28 USD)	161	60.3
High (> UGX100,000 ≈ 28 USD)	106	39.7
Religion		
Muslim	80	30.0
Christian	187	70.0

#### Young people’s perceptions of HIVST kits

For each perception item, overall agreement was calculated by combining “Strongly Agree” and “Agree” responses. Overall, participants reported positive perceptions of HIVST. A total of 59.2% agreed that HIVST kits are as accurate as conventional facility-based testing. Convenience for young people was endorsed by 65.5% of respondents. Confidentiality and privacy received the highest level of support, with 81.7% reporting agreement. In addition, 77.1% agreed that HIVST reduces stigma associated with HIV testing, and 70.4% indicated that the kits provide clear instructions. Finally, 73.1% agreed that HIVST increases access to testing among marginalized populations (Table 2).

**Table 2:** Perceptions of young people towards HIVST kits.

Variable	Strongly agree	Agree	Not sure	Disagree	Strongly disagree	Mean (SD)
HIV self-testing kits are as accurate as traditional testing methods	17.6% (47)	41.6% (111)	19.9% (53)	13.5% (36)	7.5% (20)	2.52 (1.15)
An HIV self-testing kit is a convenient option for young people	24.7% (66)	40.8% (109)	13.9% (37)	12.7% (34)	7.9% (21)	2.38 (1.21)
HIV self-testing kits offer confidentiality and privacy of HIV self-testing	57.7% (154)	24.0% (64)	10.1% (27)	5.6% (15)	2.6% (7)	1.72 (1.03)
An HIV self-testing kit would reduce the stigma associated with HIV testing	48.3% (129)	28.8% (77)	12.7% (34)	6.4% (17)	3.8% (10)	1.88 (1.09)
Self-testing kits provide enough instructions for support and guidance during the testing process	25.1% (67)	45.3% (121)	18.0% (48)	10.5% (28)	1.1% (3)	2.17 (0.96)
HIV self-testing kits are increasing access to HIV testing among marginalized populations	50.6% (135)	22.5% (60)	18.4% (49)	6.7% (18)	1.9% (5)	1.87 (1.06)

### Acceptability and utilization of HIVST kits

#### Acceptability

**Figure 1:**
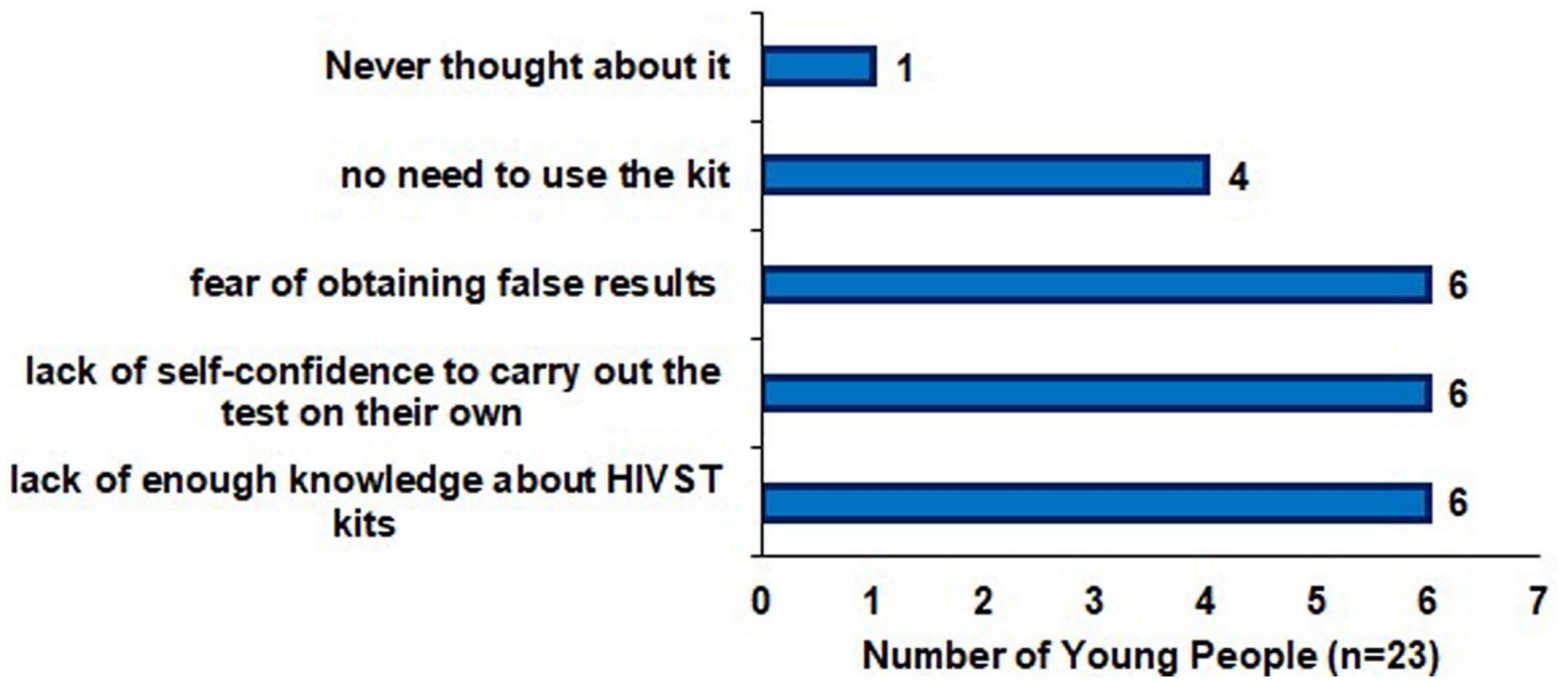
Reasons young people in the Katanga slum in Kampala were unwilling to try HIV self-testing kits.

87.6% of young people expressed willingness to try HIVST kits, while 12.4% were either unwilling or unsure. Reasons for unwillingness included insufficient knowledge about HIVST, lack of self-confidence to perform the test, and fear of obtaining inaccurate results (Figure 1). Those who were unsure (*n* = 10), most cited lack of knowledge (*n* = 7) or fear of false results (*n* = 3).

#### Utilization

**Figure 2:**
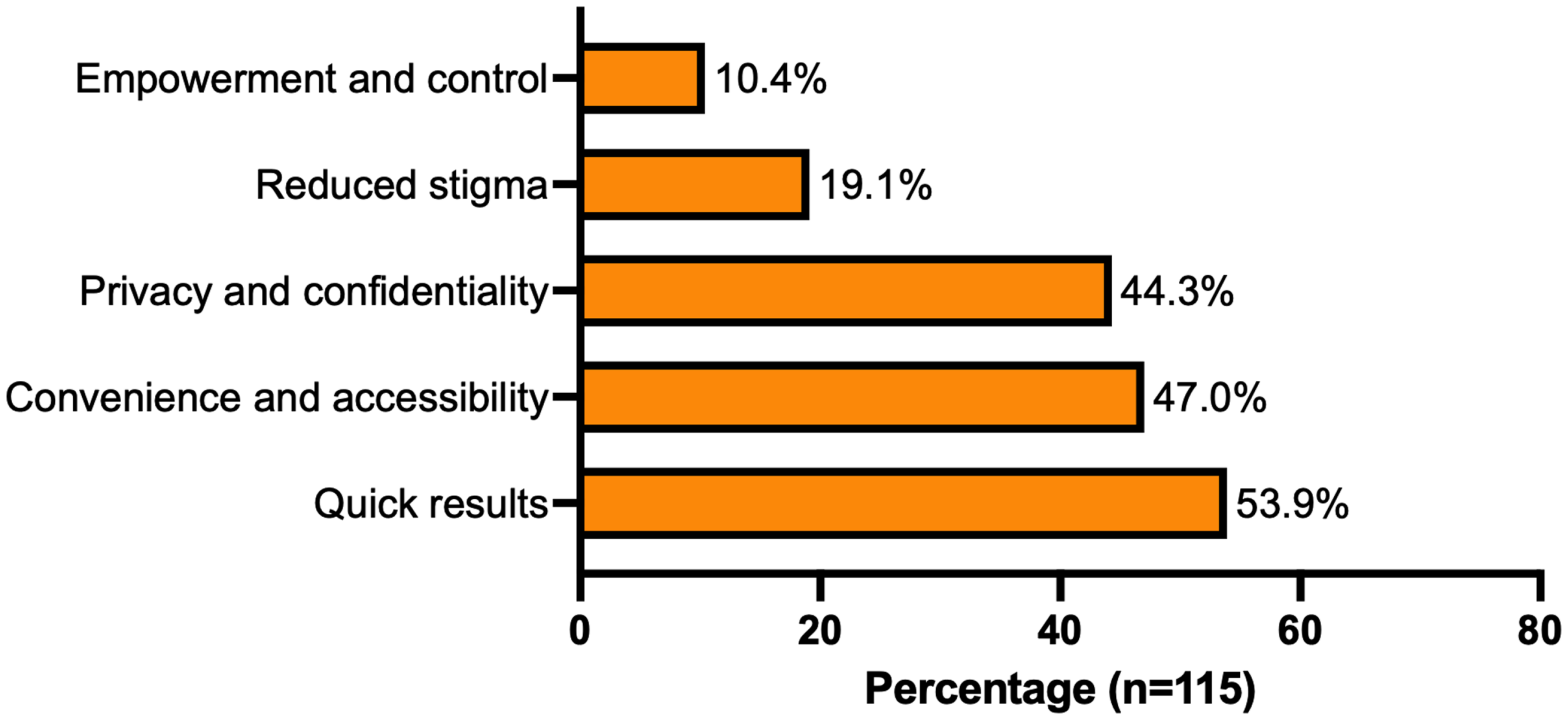
Reasons for prior use of HIVST kits among youths in Katanga slum, Kampala.

43.1% of the young people had previously used HIVST kits, 41.7% of whom reported testing every three months, 42.6% had used a kit only once, and 15.7% had not used a kit in the past year. The most frequently cited reasons for using HIVST included privacy and confidentiality, convenience and accessibility, and quick results (Figure 2).

75.7% of the young people found HIVST easy to perform, while the rest reported difficulty. 92.2% of the young people who had prior use of HIVST kits found interpretation of the test results easy, but only 62.6% sought further services or counseling after using HIVST. Contexts in which HIVST was used included regular testing to know one’s status (47%), before engaging with a new sexual partner (40%), after possible HIV exposure (12.1%), and when considering work abroad (0.9%).

### Factors associated with acceptability and utilization of HIVST kits

#### Acceptability

Participants who had ever been married had significantly higher odds of accepting HIVST (aOR 8.89, 95% CI 1.01–77.31; *p* = 0.048). Peer discussions about HIVST were also positively associated with acceptability (aOR 2.48, 95% CI 1.04–5.90; *p* = 0.040), as was perceived family support for HIVST use (aOR 2.57, 95% CI 1.07–6.23; *p* = 0.035). In contrast, financial barriers were associated with reduced likelihood of acceptability (aOR 0.27, 95% CI 0.10–0.74; *p* = 0.011) (Table 3).

**Table 3:** Factors associated with acceptability of HIVST kits.

Variable	Accepted HIVST kits *n* (%)		OR (95% CI)	*p*-value	aOR (95% CI)	*p*-value
	Yes	No				
Religion						
Christian (ref)	164 (87.7%)	23 (12.3%)	Ref	-	Ref	-
Muslim	70 (87.5%)	10 (12.5%)	0.98 (0.44–2.17)	0.964	-	-
Marital status						
Never married (ref)	186 (85.3%)	32 (14.7%)	Ref	-	Ref	-
Ever been married	48 (98.0%)	1 (2.0%)	8.30 (1.10–61.97)	**0.040**	8.89 (1.01–77.31)	**0.048**
Education status						
Low (ref)	92 (82.9%)	19 (17.1%)	Ref	-	Ref	-
High	142 (91.0%)	14 (9.0%)	2.09 (1.00–4.38)	**0.050**	1.27 (0.55–2.95)	0.577
Income status						
Low (ref)	140 (87.0%)	21 (13.0%)	Ref	-	Ref	-
High	94 (88.7%)	12 (11.3%)	1.18 (0.55–2.50)	0.676	-	-
Gender						
Female (ref)	85 (85.9%)	14 (14.1%)	Ref	-	Ref	-
Male	149 (88.7%)	19 (11.3%)	1.29 (0.62–2.71)	0.498	-	-
Duration of stay in the slum						
< 5 years (ref)	93 (84.6%)	17 (14.4%)	Ref	-	Ref	-
> 5 years	141 (89.8%)	16 (10.2%)	1.61 (0.77–3.35)	0.201	-	-
Discussed HIVST with peers						
No (ref)	80 (78.4%)	22 (21.6%)	Ref	-	Ref	-
Yes	154 (93.3%)	11 (6.7%)	3.85 (1.77–8.33)	**0.001**	2.48 (1.04–5.90)	**0.040**
Challenges accessing traditional HIV testing						
No (ref)	133 (84.2%)	25 (15.8%)	Ref		Ref	
Yes	101 (92.7%)	8 (7.3%)	2.37 (1.03–5.48)	**0.043**	1.86 (0.74–4.68)	0.186
HIVST kits readily available						
No (ref)	147 (86.5%)	23 (13.5%)	Ref	-	Ref	-
Yes	87 (89.7%)	10 (10.3%)	1.36 (0.62–2.99)	0.443	-	-
Aware of benefits of regular HIV testing						
No (ref)	21 (75.0%)	7 (25.0%)	Ref	-	Ref	-
Yes	213 (89.1%)	26 (10.9%)	2.73 (1.05–7.04)	**0.038**	0.94 (0.26–3.34)	0.922
Aware of benefits of early HIV diagnosis						
No (ref)	19 (70.4%)	8 (29.6%)	Ref	-	Ref	-
Yes	215 (89.6%)	25 (10.4%)	3.62 (1.43–9.12)	**0.006**	2.57 (0.77–8.52)	0.122
Family support for HIVST						
No (ref)	37 (71.2%)	15 (28.8%)	Ref	-	Ref	-
Yes	197 (91.6%)	18 (8.4%)	4.44 (2.05–9.58)	**0.000**	2.57 (1.07–6.23)	**0.035**
Community leaders support HIVST						
No (ref)	74 (81.3%)	17 (18.7%)	Ref	-	Ref	-
Yes	160 (90.9%)	16 (9.1%)	2.30 (1.10–4.79)	**0.027**	1.66 (0.70–3.93)	0.250
Faced financial barriers for HIVST						
No (ref)	96 (94.1%)	6 (5.9%)	Ref	-	Ref	-
Yes	138 (83.6%)	27 (16.4%)	0.32 (0.12–0.80)	**0.015**	0.27 (0.10–0.74)	**0.011**
Accessibility of traditional HIV testing						
Moderately accessible (ref)	121 (87.7%)	17 (12.3%)	Ref	-	Ref	-
Not accessible	56 (82.3%)	12 (17.7%)	0.65 (0.29–1.46)	0.303	-	-
Very accessible	57 (93.4%)	4 (6.6%)	2.00 (0.64–6.22)	0.230	-	-

Bold p-values after the OR column indicate variables selected for inclusion in the multivariable analysis, while bold p-values after the aOR column indicate statistically significant associations in the multivariable model (*p* < 0.05). HIVST: HIV self-testing; OR: Odds ratio; aOR: Adjusted odds ratio; CI: Confidence interval; Ref: reference category.

#### Utilization

Muslims were twice as likely to use HIVST as Christians (aOR 2.10, 95% CI 1.14–3.88; *p* = 0.017). HIVST peer discussions were significantly associated with utilization (aOR 3.21, 95% CI 1.71–6.04; *p* < 0.001). Perceived kit availability showed a borderline association (aOR 1.76, 95% CI 0.98–3.16; *p* = 0.057) (Table 4).

**Table 4:** Factors associated with utilization of HIVST kits.

Variable	Used HIVST kits *n (%)*		OR (95% CI)	*p-*value	aOR (95% CI)	*p*-value
	Yes	No				
Religion						
Christian (ref)	69 (36.9%)	118 (63.1%)	Ref	-	Ref	-
Muslim	46 (57.5%)	34 (42.5%)	2.31 (1.35–3.95)	**0.002**	2.10 (1.14–3.88)	**0.017**
Marital status						
Never married (ref)	87 (39.9%)	131 (60.1%)	Ref	-	Ref	-
Ever been married	28 (57.1%)	21 (42.9%)	2.00 (1.07–3.75)	**0.029**	1.42 (0.67–3.00)	0.360
Education status						
Low (ref)	40 (36.0%)	71 (64.0%)	Ref	-	Ref	-
High	75 (48.0%)	81 (52.0%)	1.64 (0.99–2.71)	**0.051**	1.27 (0.71–2.28)	0.412
Income status						
Low (ref)	64 (39.7%)	97 (60.3%)	Ref	-	Ref	-
High	51 (48.1%)	55 (51.9%)	1.40 (0.85–2.30)	**0.178**	1.22 (0.67–2.21)	0.519
Gender						
Female (ref)	41 (41.4%)	58 (58.6%)	Ref	-	Ref	-
Male	74 (44.0%)	94 (56.0%)	1.11 (0.67–1.84)	0.675	-	-
Duration of stay in the slum						
< 5 years (ref)	36 (32.7%)	74 (67.3%)	Ref	-	Ref	-
> 5 years	79 (50.3%)	78 (49.7%)	2.08 (1.25–3.45)	**0.005**	1.49 (0.84–2.68)	0.175
Discussed HIVST with peers						
No (ref)	23 (22.5%)	79 (77.5%)	Ref	-	Ref	-
Yes	92 (55.8%)	73 (44.2%)	4.32 (2.48–7.55)	**0.000**	3.21 (1.71–6.04)	**0.000**
Challenges accessing traditional HIV testing						
No (ref)	57 (36.1%)	101 (63.9%)	Ref	-	Ref	-
Yes	58 (53.2%)	51 (46.8%)	2.02 (1.23–3.31)	**0.006**	1.47 (0.82–2.65)	0.197
HIVST kits readily available						
No (ref)	59 (34.7%)	111 (65.3%)	Ref	-	Ref	-
Yes	56 (57.7%)	41 (42.3%)	2.57 (1.54–4.29)	**0.000**	1.76 (0.98–3.16)	0.057
Aware of benefits of regular HIV testing						
No (ref)	6 (21.4%)	22 (78.6%)	Ref	-	Ref	-
Yes	109 (45.6%)	130 (54.4%)	3.10 (1.20–7.85)	**0.019**	2.66 (0.76–9.31)	0.125
Aware of benefits of early HIV diagnosis						
No (ref)	4 (14.8%)	23 (85.2%)	Ref	-	Ref	-
Yes	111 (46.2)	129 (53.8)	4.95 (1.66–14.74)	**0.004**	1.00 (0.31–3.19)	0.999
Family support for HIVST						
No (ref)	19 (36.5%)	33 (63.5%)	Ref	-	Ref	-
Yes	96 (44.7%)	119 (55.3%)	1.40 (0.75–2.62)	0.290	-	-
Community leaders support HIVST						
No (ref)	31 (34.1%)	60 (65.9%)	Ref	-	Ref	-
Yes	84 (47.7%)	92 (52.3%)	1.76 (1.05–2.98)	**0.033**	1.48 (0.81–2.72)	0.204
Faced financial barriers for HIVST						
No (ref)	47 (46.1%)	55 (53.9%)	Ref	-	Ref	-
Yes	68 (41.2%)	97 (58.9%)	0.82 (0.49–1.35)	0.435	-	-
Accessibility of traditional HIV testing						
Moderately accessible (ref)	65 (47.1%)	73 (52.9%)	Ref	-	Ref	-
Not accessible	21 (30.9%)	47 (69.1%)	0.50 (0.27–0.93)	**0.028**	0.56 (0.27–1.16)	0.120
Very accessible	29 (47.5%)	32 (52.5%)	1.02 (0.63–1.24)	0.954	1.20 (0.59–2.42)	0.615

Bold p-values after the OR column indicate variables selected for inclusion in the multivariable analysis, while bold p-values after the aOR column indicate statistically significant associations in the multivariable model (*p* < 0.05). HIVST: HIV self-testing; OR: Odds ratio; aOR: Adjusted odds ratio; CI: Confidence interval; Ref: reference category.

#### Qualitative

The focus group discussion identified six key themes: privacy and confidentiality, convenience, cost and availability barriers, accuracy and trust in results, need for education and proper instructions, and psychosocial impact and support.

##### Theme one: privacy and confidentiality

Young people highlighted the importance of privacy in HIV testing. They expressed a strong preference for HIVST kits over traditional hospital testing due to concerns about confidentiality and the fear of their HIV status being exposed to the community.

*“When you use an HIVST kit, only you and your God know the result.” FGD Male 21 years.*

##### Theme two: convenience

The convenience and ease of use of HIVST kits were frequently mentioned. Young people appreciated that HIVST kits allow them to test themselves at home, avoiding the need to visit hospitals, which can be time-consuming and costly.

*“We do not see any problem with using the HIVST kits. It is much better than going to the hospital because, with an HIVST kit, you unpack it and test yourself within minutes.” FGD Female 20 years.*

##### Theme three: availability and cost barriers

While there was a high level of interest in HIVST kits, cost and availability were significant barriers. The young people noted that the kits are often expensive and not readily available in their community, which limits their utilization.

*“If the HIVST kits were free and available in plenty, I would test myself every week, but they are expensive.” FGD Male 21 years.*

*“We don’t know where to buy the HIVST kits from.” FGD Male 18 years.*

##### Theme four: accuracy and trust in results

There were mixed perceptions about the accuracy of HIVST kits. Some of the young people trusted the results from self-testing, while others preferred to confirm their results at a hospital. Concerns about the possibility of user error affecting the accuracy of the results were also raised.

*“Hospital results are more accurate because someone else who is an expert has done the test on your behalf.” FGD Female 22 years.*

*“It is better to carry out the HIVST and then go to the hospital to confirm the results.” FGD male 20 years.*

##### Theme five: need for education and proper instructions

The need for clear instructions and educational programs on how to use HIVST kits was emphasized. The young people pointed out that both pictorial and written instructions are necessary to ensure proper use, especially for the illiterate.

*“There should be both pictorial and written instructions on how to use the HIVST kits because some people might be illiterate and can’t read.” FGD male 17 years.*

##### Theme six: psychosocial impact and support

The emotional impact of receiving a positive HIV result through self-testing was a concern. Participants stressed the importance of post-test counseling to help individuals cope with their results and avoid potential negative outcomes such as suicide. The role of support systems and the availability of counseling services were deemed crucial.

*“Counselling is nice, and it should take place after self-testing. Feeling sad after getting a positive result from HIVST might result in suicide due to many thoughts and stress.” FGD female 22 years.*

## Discussion

This study examined the perception, acceptability, and utilization of HIVST kits among young people in the Katanga Slum of Kampala. Findings indicate that although the acceptability of HIVST is high, actual utilization remains relatively low, and young people generally have positive perceptions toward HIVST kits. The former reinforces earlier reviews from Sub-Saharan Africa (SSA), where acceptability often surpassed actual use (18). Key barriers included concerns about pre-and post-test counseling, reliability of test results, and the cost of kits. These barriers have also been widely reported in African settings (9, 18). Moreover, the absence of post-test counseling discouraged some youth from using HIVST kits, as seen in studies from Malawi and Uganda (8, 19). Most participants perceived HIVST as convenient, private, and capable of reducing stigma, findings consistent with reality and previous studies from Namibia, Nigeria, and Ethiopia that highlight similar favorable attitudes among youth toward HIVST kits due to their discretion and user autonomy (10, 18, 20). Acceptability was significantly associated with marital status, peer discussions, family support, and lack of financial barriers. Those who have ever been married were more likely to report acceptability, possibly reflecting a heightened sense of responsibility or consolidated social support structures. Peer engagement appears to be a powerful influence, consistent with prior evidence showing that peer-led models can boost both HIVST demand and uptake among young women in Uganda and broader SSA contexts (21, 22). Similarly, perceived family support underlined the social ecosystem’s role in facilitating acceptability. Financial constraints strongly reduced acceptability, mirroring multiple studies in Africa where cost has been identified as a major obstacle to HIVST uptake in marginalized populations (18, 23). Regarding utilization, Muslim participants had more than twice the odds of using HIVST compared to Christians, possibly reflecting the influence of community-level religious engagement and inter-faith health promotion efforts in Uganda (24). Peer discussions remained a strong predictor of use (aOR 3.21), supporting the effectiveness of peer distribution and education in increasing HIVST uptake (25, 26). Perceived availability of kits was marginally associated with use, pointing to the importance of supply continuity in translating acceptability into actual testing behavior. HIV self-testing Kits are available for free in public health centers and at low cost in pharmacies (1 USD) to increase accessibility; however (27), many youths in informal settlements such as Katanga still point out cost as a barrier to utilization of HIVST. This explains participants’ perceptions that kits are difficult to obtain or costly, perceptions that may affect their acceptability and utilization, which is not the reality.

These findings highlight that even high acceptability does not guarantee use; structural and social enablers are crucial for bridging this gap. The role of peer networks and supportive social environments emerged as central to both acceptability and uptake, while financial constraints and inconsistent access continue to hinder broader HIVST implementation. Given these concerns, practical strategies such as community-based distribution through youth centers, integration of HIVST into slum outreach programs, subsidization of kits in private drug shops, and strengthening linkage-to-care pathways could directly address participants’ needs.

### Strengths and limitations

This study addresses a locally relevant public health issue using a mixed-methods design, enhancing validity. It maintained strong ethical standards. The real-world setting, engagement of local youth, and inclusion of a hard-to-reach population increase contextual relevance and policy applicability. However, the single-site focus may limit generalizability. Self-reported data may introduce bias. The qualitative component consisted of only one focus group discussion, which, while providing valuable contextual insights, was insufficient to achieve thematic saturation; therefore, some perspectives may not have been fully captured.

## Conclusion

Despite the high acceptability of HIVST among young people in Kampala’s urban unplanned settlements, utilization remains low due to financial barriers, limited kit availability, and gaps in supportive systems. Peer-led outreach, community engagement, and subsidized kit distribution could bridge the gap between acceptability and use. Future interventions should leverage youth networks, address affordability, and ensure consistent supply to expand the reach and impact of HIVST among underserved urban populations.

## Data Availability

The raw data supporting the conclusions of this article will be made available by the authors, without undue reservation.

## References

[1] UNAIDS. Uganda. 2023 (Accessed Oct 9, 2024). Available online at: https://www.unaids.org/en/keywords/uganda

[2] Thomas L Vearey J Mahlangu P. Making a difference to health in slums: an HIV and African perspective. Lancet. (2011) 377:1571–2. doi: 10.1016/S0140-6736(11)60642-9, 21550480

[3] Ortblad K Kibuuka Musoke D Ngabirano T Nakitende A Magoola J Kayiira P . Direct provision versus facility collection of HIV self-tests among female sex workers in Uganda: a cluster-randomized controlled health systems trial. PLoS Med. (2017) 14:e1002458. doi: 10.1371/journal.pmed1002458, 29182634 PMC5705079

[4] Benyumiza D Amongin JF Ochaba I Adupa M Abuch N Banula CB . Factors associated with utilization of HIV testing services among adolescents aged 10-19 years in Lira District, northern Uganda: a cross-sectional study. Biomed Res Int. (2021) 2021:1–7. doi: 10.1155/2021/9568148, 34423039 PMC8376469

[5] Johnson CC Kennedy C Fonner V Siegfried N Figueroa C Dalal S . Examining the effects of HIV self-testing compared to standard HIV testing services: a systematic review and meta-analysis. J Int AIDS Soc. (2017) 20:1–3. doi: 10.7448/IAS.20.1.21594, 28530049 PMC5515051

[6] Stekler J Wood B Ballenger C. Arguments for and against HIV self-testing. HIV/AIDS - Research and Palliative Care. (2014) 6:117–26. doi: 10.2147/HIV.S49083, 25114592 PMC4126574

[7] Hatzold K Gudukeya S Mutseta MN Chilongosi R Nalubamba M Nkhoma C . HIV self-testing: breaking the barriers to uptake of testing among men and adolescents in sub-Saharan Africa, experiences from STAR demonstration projects in Malawi, Zambia and Zimbabwe. J Int AIDS Soc. (2019) 22:e25244. doi: 10.1002/jia2.25244, 30907505 PMC6432104

[8] Choko AT Desmond N Webb EL Chavula K Napierala-Mavedzenge S Gaydos CA . The uptake and accuracy of Oral kits for HIV self-testing in high HIV prevalence setting: a cross-sectional feasibility study in Blantyre, Malawi. PLoS Med. (2011) 8:e1001102. doi: 10.1371/journal.pmed.1001102, 21990966 PMC3186813

[9] Njau B Covin C Lisasi E Damian D Mushi D Boulle A . A systematic review of qualitative evidence on factors enabling and deterring uptake of HIV self-testing in Africa. BMC Public Health. (2019) 19:1289. doi: 10.1186/s12889-019-7685-1, 31615461 PMC6794839

[10] Mhango M Dubula-Majola V Mudadi LS. Knowledge, attitudes and perceptions about HIV self-testing amongst college students in Namibia. F1000Res. (2022) 11:11. doi: 10.12688/f1000research.55670.1

[11] Volk JE Lippman SA Grinsztejn B Lama JR Fernandes NM Gonzales P . Acceptability and feasibility of HIV self-testing among men who have sex with men in Peru and Brazil. Int J STD AIDS. (2016) 27:531–6. doi: 10.1177/0956462415586676, 25971262 PMC4643427

[12] Oduetse OK Nkomo B Majingo N Mashalla Y Seloilwe E. Perceptions and attitudes towards acceptability of HIV self-testing among female sex workers in Selibe Phikwe, Botswana. Afr J AIDS Res. (2019) 18:192–7. doi: 10.2989/16085906.2019.1638427, 31469045

[13] Stevens DR Vrana CJ Dlin RE Korte JE. A global review of HIV self-testing: themes and implications. AIDS Behav. (2018) 22:497–512. doi: 10.1007/s10461-017-1707-8, 28155039 PMC5910655

[14] Omulo G Muhsin M Kasana I Nabaterega R. A proposal for empowering slum dwellers as a viable way of addressing urbanization challenges in Katanga slum, Kampala, Uganda. Environ Eng Res. (2017) 22:432–8. doi: 10.4491/eer.2017.013

[15] Ssemugabo C Nalinya S Lubega GB Ndejjo R Musoke D. Health risks in our environment: urban slum youth’ perspectives using photovoice in Kampala, Uganda. Sustainability. (2020) 13:248. doi: 10.3390/su13010248

[16] Baluku JB Anguzu G Nassozi S Babirye F Namiiro S Buyungo R . Prevalence of HIV infection and bacteriologically confirmed tuberculosis among individuals found at bars in Kampala slums, Uganda. Sci Rep. (2020) 10:13438. doi: 10.1038/s41598-020-70472-6, 32778729 PMC7417543

[17] Babatunde AO Agboola P Babatunde Y Ilesanmi EB Ayodele H Ezechi OC. Assessment of knowledge and acceptability of HIV self-testing among students of selected universities in Southwest Nigeria: an online cross-sectional study. Pan Afr Med J. (2022) 43:43. doi: 10.11604/pamj.2022.43.94.31741, 36660087 PMC9816886

[18] Zeleke EA Stephens JH Gesesew HA Gello BM Ziersch A. Acceptability and use of HIV self-testing among young people in sub-Saharan Africa: a mixed methods systematic review. BMC Primary Care. (2024) 25:369. doi: 10.1186/s12875-024-02612-0, 39407123 PMC11475945

[19] Okoboi S Twimukye A Lazarus O Castelnuovo B Agaba C Immaculate M . Acceptability, perceived reliability and challenges associated with distributing HIV self-test kits to young MSM in Uganda: a qualitative study. J Int AIDS Soc. (2019) 22:e25269. doi: 10.1002/jia2.25269, 30932364 PMC6441924

[20] Obiezu-Umeh C Gbajabiamila T Ezechi O Nwaozuru U Ong JJ Idigbe I . Young people’s preferences for HIV self-testing services in Nigeria: a qualitative analysis. BMC Public Health. (2021) 21:67. doi: 10.1186/s12889-020-10072-1, 33413246 PMC7792110

[21] Olum R Geng EH Kitutu FE Musoke PM. Feasibility, acceptability and preliminary effect of a community-led HIV self-testing model among adolescent girls and young women in rural northern Uganda: a quasi-experimental study protocol. Implement Sci Commun. (2024) 5:56. doi: 10.1186/s43058-024-00596-7, 38773505 PMC11110295

[22] Nakalega R Mukiza N Menge R Kizito S Babirye JA Kuteesa CN . Feasibility and acceptability of peer-delivered HIV self-testing and PrEP for young women in Kampala, Uganda. BMC Public Health. (2023) 23:1163–12. doi: 10.1186/s12889-023-16081-0, 37322510 PMC10273744

[23] Segawa I Bakeera-Kitaka S Ssebambulidde K Muwonge TR Oriokot L Ojiambo KO . Factors associated with HIV self-testing among female university students in Uganda: a cross-sectional study. AIDS Res Ther. (2022) 19:59. doi: 10.1186/s12981-022-00484-x, 36457098 PMC9713199

[24] Kagimu M Guwatudde D Rwabukwali C Kaye S Walakira Y Ainomugisha D. Inter-religious cooperation for HIV prevention in Uganda: a study among Muslim and Christian youth in Wakiso District. Religion. (2011) 2:707–28. doi: 10.3390/rel2040707

[25] Matovu JKB Nambuusi A Nakabirye S Wanyenze RK Serwadda D. Formative research to inform the development of a peer-led HIV self-testing intervention to improve HIV testing uptake and linkage to HIV care among adolescents, young people and adult men in Kasensero fishing community, Rakai, Uganda: a qualitative study. BMC Public Health. (2020) 20:1582–16. doi: 10.1186/s12889-020-09714-1, 33081735 PMC7576713

[26] Matovu JKB Namwama AT Kemigisha L Taasi G Nakabugo J Wandabwa J . Feasibility, acceptability and preliminary effects of a social network-based, peer-led HIV self-testing intervention among men in two Ugandan fishing communities, 2022. Arch Public Health. (2025) 83:23–14. doi: 10.1186/s13690-025-01511-9, 39849572 PMC11761786

[27] Nsereko GM Kobusingye LK Musanje K Nangendo J Nantamu S Baluku MM. Self-testing knowledge and beliefs on HIV self-testing use in Central Uganda. PLoS Glob Public Health. (2024) 4:e0002869. doi: 10.1371/journal.pgph.0002869, 38865354 PMC11168646

